# Usefulness of Sweat Management for Patients with Adult Atopic Dermatitis, regardless of Sweat Allergy: A Pilot Study

**DOI:** 10.1155/2017/8746745

**Published:** 2017-01-22

**Authors:** Sakae Kaneko, Hiroyuki Murota, Susumu Murata, Ichiro Katayama, Eishin Morita

**Affiliations:** ^1^Department of Dermatology, Shimane University Faculty of Medicine, 89-1 Enya-cho, Izumo, Shimane 693-8501, Japan; ^2^Department of Dermatology, Graduate School of Medicine, Osaka University, 2-2 Yamadaoka, Suita, Osaka 565-0871, Japan

## Abstract

*Background*. Sweat is an aggravating factor in atopic dermatitis (AD), regardless of age. Sweat allergy may be involved in AD aggravated by sweating.* Objective.* We investigated whether sweat exacerbates adult AD symptoms and examined the extent of sweat allergy's involvement.* Method.* We asked 34 AD patients (17 men, 17 women; mean age: 27.8 years) to record the extent to which sweat aggravated their symptoms on a 10-point numerical scale. Participant responses were compared with histamine release tests (HRT). Furthermore, 24 of the patients received instructions on methods of sweat management, and their outcomes were evaluated on a 10-point scale.* Results. *Sweat HRT results were class ≥ 2 in 13 patients, but HRT results were not correlated with the patients' self-assessments of symptom aggravation by sweat. One month after receiving sweat management instructions, a low mean score of 4.6 was obtained regarding whether active sweating was good, but a high mean score of 7.0 was obtained in response to whether the sweat management instructions had been helpful.* Conclusion*. Our investigation showed that patients' negative impressions of sweat might derive from crude personal experiences that are typically linked to sweating. Sweat management for patients with adult atopic dermatitis was extremely useful regardless of sweat allergy.

## 1. Introduction

Atopic dermatitis (AD) is a chronic relapsing eczematous skin disease characterized by pruritus and inflammation and is accompanied by cutaneous physiological dysfunction [1]. Numerous AD-triggering factors are known, such as irritants, aeroallergens, food, microbial organisms, and sweat/sweating [2]. Skin testing with autologous sweat is positive in most patients with AD [3, 4], and the clinical symptoms of children with AD improve significantly during the summer if they take showers at school [5, 6]. Semipurified sweat antigen from normal adults induced histamine release from the basophils of 77% of patients with AD and 66% of patients with cholinergic urticaria in Japan [7, 8]. The histamine release induced by semipurified sweat antigen is mediated by specific immunoglobulin E (IgE) [7, 8]. Sweat histamine release tests (HRTs; Allerport®, Kyowa Medex Co., Ltd., Tokyo, Japan) are covered by the Japanese health insurance system. The main sweat antigen is MGL_1304, produced as a minor immunological antigen of* Malassezia globosa *with posttranslational modification, cleaved, and secreted as a 17 kDa major histamine-releasing sweat* (Malassezia)* antigen [9].

In the present study, we investigated whether sweat plays a role in exacerbating adult AD symptoms and examined the extent to which sweat allergy is involved.

## 2. Materials and Methods

### 2.1. Subjects

All patients were recruited from Shimane University Hospital between April 2012 and March 2014 before they start sweating. We enrolled 34 patients with AD that was diagnosed following the Japanese Dermatological Association's criteria. The 34 enrolled patients included 17 men and 17 women (mean age, 27.8 years; mean serum IgE, 7544 IU/mL) who received an HRT (Allerport, Kyowa Medex Co., Ltd., Tokyo, Japan). Histamine release percentage was (antigen specific histamine release amount – nonspecific histamine release amount)/(total histamine release amount − nonspecific histamine release amount) × 100. HRT class 0 does not exceed histamine release cutoff (histamine release percentage 20%) stimulated by any antigen concentration sample. HRT class 1 exceed histamine release cutoff stimulated by the darkest antigen concentration sample. HRT class 2 exceed histamine release cutoff stimulated by the darkest antigen sample and second dark antigen concentration. HRT class 3 exceed histamine release cutoff stimulated by the darkest antigen sample, second dark antigen, and third dark antigen concentration. HRT class 4 exceed histamine release cutoff stimulated by the thinnest antigen concentration sample. Eczema Area and Severity Index (EASI) developed by the EASI Evaluator Group (maximum 72 points) is used in this study [1].

### 2.2. Study Design

We asked the 34 AD patients to record the extent to which sweat aggravated their symptoms. The participants recorded their responses using a 10-point numerical scale, on which a score of 10 indicated that sweat was the greatest aggravating factor. Participant responses were compared with the classes of results obtained from the HRT of blood samples.

In a second component to this study, we also instructed 24 of the patients on methods of dealing with sweat (sweat management). We subsequently evaluated the outcomes of this instruction on a 10-point numerical scale. This study was approved by the ethical committee of Shimane University and the dean of the Faculty of Medicine (approval number 1746).

In the course of our sweat management instructions, we explained that it was fine to sweat profusely but additionally requested that patients do at least one of the following: (1) shower at least once during the day (as soon as they sweat, not since they came home), (2) wash the affected areas of their skin with water, (3) apply wet wipes made of soft dough to irritated parts of their skin, and/or (4) change their clothes when they became soaked with sweat. One month after this sweat management instruction, its outcomes were determined via a questionnaire that used a 10-point numerical scale.

### 2.3. Statistical Analysis

Spearman's rank correlation test was used to compare variables between groups. All analyses were performed using STATA version 9 (StataCorp, College Station, TX, USA). A *p* value <0.05 was considered statistically significant. Continuous values are reported as means ± standard deviations.

## 3. Results

**Figure 1:**
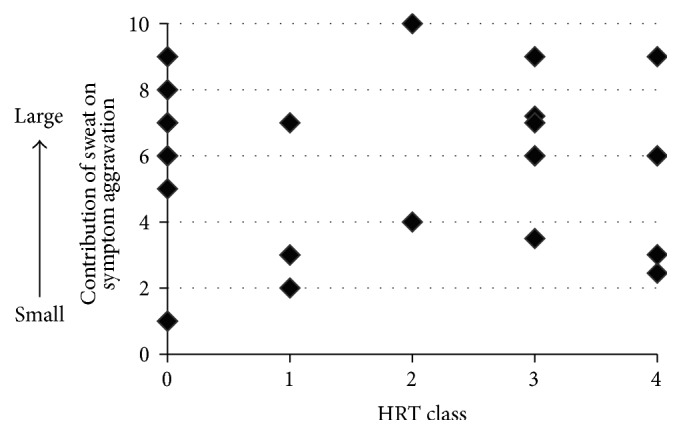
The relationship between patients' perceived symptom aggravation due to sweat and histamine release test (HRT) results (*N* = 32). Spearman's rank correlation coefficient revealed no correlations (rs = −0.102, *p* = 0.286).

In the HRT positive control test (anti-IgE antibody), 2 of the 34 participants tested negative and were excluded from the analysis; both these participants were women. A mean sweat HRT class of 1.44 ± 1.61 was recorded. During treatment, patients were asked to respond to the following question on a scale of 1–10 with a score of 10 indicating the greatest degree of aggravation: “To what extent does sweat aggravate your symptoms?” The mean of the patient scores was 5.85 ± 2.43. Blood was also collected, and correlations between the original HRT results for sweat and patient responses were tested using Spearman's rank correlation coefficient. As can be seen in Figure 1, no correlation was observed.

Subsequently, 24 of the patients received guidance about the importance of sweating as a bodily function, having the opportunity to sweat on a daily basis, and taking measures to prevent excess sweat on the skin. Regarding the patients' motivations to sweat on a daily basis, 8 of the 24 participants (one-third of the group) consciously worked up a sweat. With regard to avoiding excess sweat on the skin, 13 patients took showers, 4 washed the affected areas with water, and 7 used wipes on easily aggravated areas.

**Figure 2:**
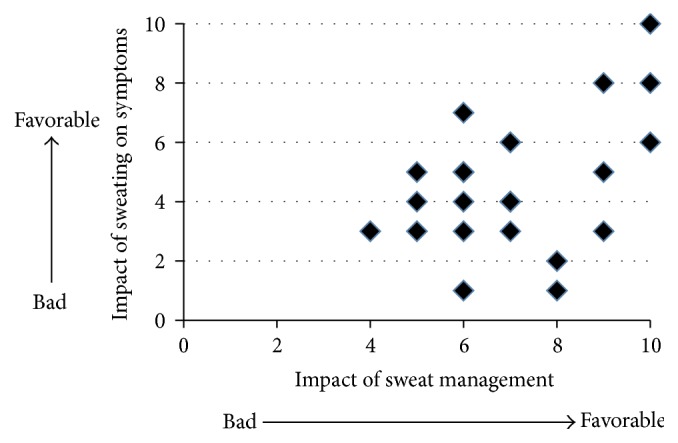
The relationship between perception of sweat and the degree of helpfulness provided by sweat management instructions at 1 month after the instructions (*N* = 24). Spearman's rank correlation test showed a strong correlation between these values (rs = 0.348, *p* = 0.0475).

One month after education on sweat management was provided, patients were asked to rate the following two statements. The first statement was “It was good to sweat,” for which a mean response score of 4.63 ± 2.20 was recorded. The second statement was “The countermeasures for sweating were helpful,” for which the mean score was 7.04 ± 1.73. Spearman's rank correlation test showed a positive correlation between these values (Figure 2). But there was no significant relation of EASI between before and after education on sweat management (*N* = 24: before 10.8 ± 4.8, after 10.7 ± 5.2). The reason might be that patients were mild cases and observation period was short.

**Figure 3:**
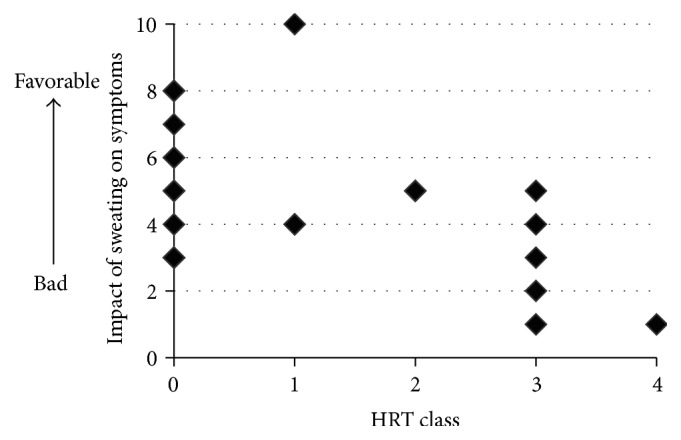
The relationship between human sweat histamine release test (HRT) class and the perception of sweat at 1 month after sweat management (*N* = 23). Spearman's rank correlation test indicated a significant correlation (rs = −0.411, *p* = 0.0271).

Furthermore, a negative correlation was found between the patients' impressions of sweating and the results of the sweat HRT (Figure 3). Patients with high HRT results (positive reaction against semipurified protein derived from healthy adults) did not have a favorable impression of sweating. However, many patients still thought that our sweating education was helpful (Figure 2), and no correlation was found between HRT class and patients' impressions of the measures against excess sweat on the skin (Figure 1).

## 4. Discussion

Patients may often be directed to avoid sweating based on the belief that sweat is an exacerbating factor for AD. Sweating plays key roles in skin homeostasis, antimicrobial [10], and moisturizing effects [11] and in skin surface pH regulation [12]. Skin care with daily showering at an elementary school was found to be effective for the treatment of atopic dermatitis [6]. However, few studies have evaluated the impact of sweat education on the awareness of patients with AD. Therefore, we conducted a pilot study to investigate the impact of education on sweating. We found no correlation between the result of HRTs against sweat antigen and the degree of a bad impression of sweat. Additionally, there was no evident correlation between the degree of a bad impression of sweat and the self-perceived extent of sweating after sweat management (Spearman's rank correlation test: rs = 0.0323, *p* = 0.438). Therefore, this does not indicate that excess sweating aggravates the symptom of AD. We also analyzed HRTs against sweat in the group that received sweat management education. The measurement results of HRTs were not affected by receiving sweat management instructions (Spearman's rank correlation test: rs = 0.0131, *p* = 0.498). In fact, patients with a bad impression of sweat showed high HRT results.

However, many patients still thought that taking measures against excess sweating was good. Furthermore, no correlation was found between the results of the HRT and patients' favorable impression of countermeasures against sweating. Thus, we concluded that it was worthwhile to offer proper guidance about sweat to patients with AD. Unexpectedly, several subjects in our study are likely to think that sweating is good for relieving their skin symptoms. This might further be interpreted as suggesting that sweating could be a mitigating factor in some cases, rather than an aggravating factor.

In addition, we administered a separate questionnaire survey to patients. In this questionnaire, patients were asked, “Have you received guidance on what you should do after sweating?” No bad impressions were found in taking measures to avoid sweat on the skin. We found that three-fourths of those surveyed have in fact been given such advice [13]. This result also indicates that patient guidance for sweating management is quite important.

In general, sweat has been thought to aggravate AD by allergic reaction against the* Malassezia* antigen or by impaired skin homeostasis due to decreased sweating. Our study found that patients' impression of “sweat is bad” might have come from crude personal experiences which are ordinarily linked with sweating (e.g., associations between sweating and hot environments, exercise, and nervousness).

## 5. Conclusions

Sweat management for patients with adult atopic dermatitis was extremely useful, regardless of sweat* (Malassezia)* allergies or bad impression for sweating.
